# Zunyimycin C enhances immunity and improves cognitive impairment and its mechanism

**DOI:** 10.3389/fcimb.2022.1081243

**Published:** 2022-12-12

**Authors:** Xuemei Wang, Zexin Li, Rui Sun, Xueli Li, Ruirui Guo, Xiangyi Cui, Bingxin Liu, Wujuan Li, Yi Yang, Xiaoyu Huang, Hanlin Qu, Chen Liu, Zhuoling Wang, Yuhong Lü, Changwu Yue

**Affiliations:** ^1^ Yan’an Key Laboratory of Microbial Drug Innovation and Transformation, School of Basic Medicine, Yan’an University, Yan’an, Shaanxi, China; ^2^ Shaanxi Key Laboratory of Chemical Reaction Engineering, College of Chemistry and Chemical Engineering, Yan’an University, Yan’an, Shaanxi, China; ^3^ Shaanxi Institute of Basic Sciences (Chemistry and Biology), Northwestern University, Xi’an, Shaanxi, China

**Keywords:** Alzheimer’s disease, zunyimycin C, cognitive impairment, behavior, immunological enhancement

## Abstract

This study aimed to explore the efficacy of zunyimycin C in the immunological enhancement of hypoimmune mice and improvement of cognitive impairment in a mice model of Alzheimer’s disease (AD). Zunyimycin C was administered intranasally to interfere with AD mouse models or gavage to hypoimmune animals. Results of the Morris water maze (MWM) showed that zunyimycin may improve the learning and memory abilities of the AD mice model. The results of differential expression analysis of mRNA levels of inflammatory factors and pathways in brain tissues of the AD mouse model suggested that differential expression was more obvious under Zun-Int L. Western blot revealed that the relative expression of glial fibrillary acidic protein in the brain tissue of the AD mouse model in the Zun-Pre group was significantly higher than that in the other groups, and the difference was statistically significant. The relative expression of interleukin (IL)-6 protein in the brain tissue of mice in the low-dose intervention group was significantly lower than that in the other groups, and the difference was statistically significant. As for hypoimmune animals, short chain fatty acids (SCFAs) assay and intestinal flora assay results showed that zunyimycin C may change intestinal flora diversity and SCFA biosynthesis. The prophylactic administration of zunyimycin C could not inhibit acute neuroinflammation in AD mice. Zunyimycin C may participate in the immune response by activating the Ras-Raf-MEK-ERK signaling pathway to stimulate microglia to produce more inflammatory factors. Zunyimycin C may inhibit autophagy by activating the PI3K-AKT-mTOR signaling pathway, promote cell survival, mediate neuroprotective effects of reactive microglia and reactive astrocytes, and reduce IL-1β in brain tissue and IL-6 secretion, thereby attenuating neuroinflammation in AD mice and achieving the effect of improving learning and memory impairment. Zunyimycin C may play a role in immunological enhancement by changing intestinal flora diversity and SCFAs.

## 1 Introduction

Alzheimer’s disease (AD) is one of the most common degenerative diseases of the central nervous system; its clinical manifestations are mainly memory loss, weakened behavioral ability, language ability loss, and cognitive impairment, causing a heavy burden to the family and society (Lane et al., 2015; Tatulian, 2022; Zhang et al., 2022; Wang et al., 2022). According to the first “Report on the Family Survival Status of Alzheimer’s Disease Patients” in China, as of 2019, more than 10 million patients have AD in China, accounting for about 25% of the world’s total patients with AD (Di Carlo et al., 2002). By 2050, the proportion of patients with AD over 80 years old will be close to 50%, which will become the main population of patients with AD (Andersen et al., 1999; Eratne et al., 2018). Studies have shown that the incidence of AD increases exponentially with age (Eratne et al., 2018). The onset of the disease is insidious ranging from mild cognitive impairment to severe cognitive impairment, and the duration of the disease varies from about 5 years to more than 10 years (2022; Bulgart et al., 2020). Symptoms are strongly pronounced in patients after mental stimulation, other diseases of the body, or fractures. The pathogenesis of AD is associated with multiple factors, including familial inheritance, β-amyloid(Aβ) deposition, microtubule-associated protein Tau (Tau) hyperphosphorylation, neuroinflammation, oxidative stress, and intestinal bacteria disorder (Chen et al. (2017); Rodrigo et al., 2013; Frigerio and Strooper, 2016).

For AD, current clinically used drugs include cholinesterase inhibitors donepezil, ristigmine, galantamine, and glutamic acid N-methyl-D-aspartic acid receptor antagonist memantine hydrochloride. In 2019, China launched a new domestic drug targeting the brain–gut axis for AD treatment, namely, Mannat sodium capsule (also known as GV-971) (Cacabelos and Torrellas, 2014; Gąsiorowski et al., 2022). In 2021, the United States Food and Drug Administration approved aducanumab, an antibody against Aβ, for the treatment of AD. Treatment with these drugs has achieved certain clinical results (Lancet, 2016), but they cannot solve the problem of irreversible disease (Briggs et al., 2016).

**Figure 1:**
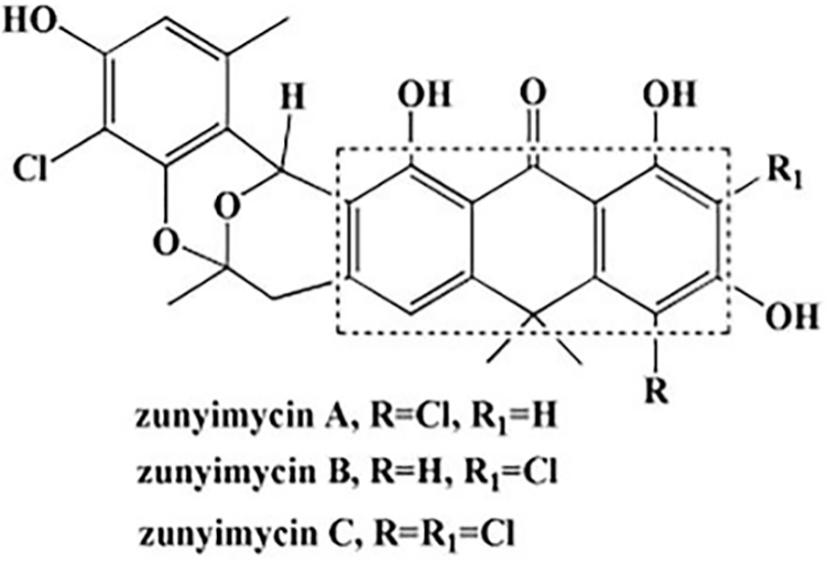
Chemical structures of zunyimycins.

Zunyimycins (**Figure 1**) are a series of halogenated type II polyketide antibiotics, which we have isolated the chlorinated modified compounds of the type II polyketide BE24566B and its chloroderivatives called zunyimycin A, zunyimycin B, zunyimycin C and chloroanthrabenzoxocinone from the fermentation solid culture of Streptomyces sp. FJS31-2. As a novel antibiotic, the application of Zunyimycin is still under the preclinical investigation stage (Lü et al., 2016). The results of previous studies showed that zunyimycin C exerts different degrees of inhibitory effects on pathogenic bacteria such as methicillin-resistant Staphylococcus aureus (MRSA) and *Enterococcus faecalis (* Lü et al., 2017). Zunyimycin C has a strong killing effect on Hep G2, MKC45, and A549 tumor cells.

The previous proteomic analysis of zunyimycin C-treated cells in our group found that 5 AD-related proteins were differentially expressed, and expression profiling analysis revealed that 26 AD-related genes were differentially expressed, of which 19 genes were downregulated and the expression of 7 genes was upregulated. Therefore, zunyimycin C may have anti-AD activity.

C57BL/6J mice were given intracerebroventricular injection of Aβ_1-42_ oligomers to construct an AD mouse model (Chen et al., 2013; Hasan et al., 2021). This AD model is often used to study the therapeutic effects of drugs, the effects on the occurrence and development of diseases, and the effects on learning and memory ability (Kim et al., 2016; Ahmad et al., 2021). Studies have shown that intranasal administration can directly deliver drugs to the central nervous system across the blood-brain barrier and brain-cerebrospinal fluid barrier (Martins et al., 2019; Wang et al., 2019), with the advantages of non-invasiveness, painlessness, rapid onset of action, and reduction of systemic side effects (Illum, 2000; Jansson and Björk, 2002).

This study mainly explored the anti-AD activity and mechanism of zunyimycin C from the animal level to provide a theoretical reference for zunyimycin C in the treatment of AD and determine its mechanism, as well as provide a theoretical basis for the further development of zunyimycin C.

## 2 Material and methods

### 2.1 Animals

Male C57BL/6 mice (7 weeks old weighing 20–22 g) were used in this study. The mice were purchased from Jiangsu Huachuang Xinnuo Pharmaceutical Technology Co., Ltd. (Jiangsu, China, SCXK (Su) 2020-0009). The mice were housed with the Animal Experiment Center of Yan’an University and given free access to food and water under temperature- and humidity-controlled conditions with a 12 h light/dark cycle. Experiments started after 7 days of adaptive feeding. All procedures were approved by the Animal Ethics Committee, Yan’an University, China, and complied with the National Institutes of Health Guidelines for the Care and Use of Laboratory Animals.

### 2.2 *In vivo* interventions for the AD mouse model

Mice were randomly divided into nine groups (**Table 1**). The AD mouse model was established by injecting Aβ_1-42_ oligomers (Aβ_1-42_Os) (10 µg, GenScript Probio Co., Ltd., Nanjing, China) into the lateral ventricle (AP −0.2, ML ±1.0, DV −2.5) as previously described. Before Aβ_1-42_O injection, mice were continuously treated with zunyimycin C (14 mg/kg, nasal instillation) for 21 days to study the preventive effect of zunyimycin C on AD. Other groups were continuously treated with different doses of zunyimycin C, donepezil, benfotiamine, or vehicle for 21 days. Donepezil and benfotiamine were purchased from Shanghai Aladdin Biochemical Technology Co., Ltd. (Shanghai, China). Behavioral tests were assessed 21 days after intervention. Mice were sacrificed after behavioral testing, and the brains of mice were collected. The left brain was used to measure Il-1β, Tnf-α, IFN-γ, Iba1, Creb, Pparγ, Mek1, Akt, and Gapdh by reverse transcription polymerase chain reaction (RT-PCR). The right brain was used to measure the glial fibrillary acidic protein (GFAP), CD163, interleukin (IL)-6, IL-1β, and GAPDH by Western blot.

**Table 1:** Mice grouping information.

Group	Drug	Intervene (Before or after modeling)	Way of administration	Modeling or not	n
Zun-Pre	Zunyimycin C, 14 mg/kg/d	21days before	Nasal cavity	Modeling	7
Zun-Int L	Zunyimycin C,7 mg/kg/d	After	Nasal cavity	Modeling	7
Zun-Int M	Zunyimycin C, 14 mg/kg/d	After	Nasal cavity	Modeling	7
Zun-Int H	Zunyimycin C, 21 mg/kg/d	After	Nasal cavity	Modeling	7
Donepezil	Donepezil, 5 mg/kg/d	After	Gavage	Modeling	7
Benfotiamine	Benfotiamine, 100 mg/kg/d	After	Gavage	Modeling	7
Benfotiamine +Zun-Int M	Benfotiamine, 100 mg/kg/d and zunyimycin C, 14 mg/kg/d	After	Nasal cavity and gavage	Modeling	7
Aβ42	—	—	—	Modeling	7
Vehicle	—	—	—	Not	7

### 2.3 Morris water maze test

The Morris water maze (MWM) was performed with reference to the previous literature (Othman et al., 2022). An MWM analysis system (Zhongshi DiChuang Technology Co., Ltd., Beijing, China) was used for this experiment. The MWM was carried out in a circular pool (120 cm in diameter and 40 cm high) filled with water (the height of the water was 30 cm). The pool was divided into four quadrants. A platform with a diameter of 8 cm was located in the third quadrant, 1 cm underwater, and the water temperature was controlled at 22°C. Mice were trained four times a day at the same time period, with each time from a different quadrant, for 5 consecutive days. Mice were placed in the pool for 60 s during training. If the mouse successfully found the platform, it was allowed to rest on the platform for 10 s. If the mouse did not successfully find the platform, it was guided to the platform and rested for 10 s. On the 6th day, the platform was removed, and the mice were placed into the pool from the diagonal quadrant of the original platform and allowed to swim in the pool for 60 s. The animal behavior analysis system was used to record the animal’s on-stage latency, distance before on-stage, and swimming speed.

### 2.4 SCFA assay

Twenty four mice were randomly divided into three groups, namely, blank (KB), model group (CTX), and zunyimycin C group. Mice were intraperitoneally injected with CTX (100 mg/kg) once a day for 3 days to build an immunosuppressive mouse model, whereas mice in the KB group were intraperitoneally injected with normal saline (NS) as control. From the fourth day, mice were given normal saline by gavage in the KB and CTX groups, and other mice were given the drug for 15 days. The feces were collected with sterile centrifuge tubes and stored at −80 °C. SCFA was determined by Shanghai LuMing Biotechnology Company *via* LC-MS-MS.

SCIEX OS-MQ software (Sciex, USA) was employed to identify and integrate MRM transitions with default parameters. Abscissa of the extracted ion current diagram (XIC) of each substance was the retention time (RT) of metabolite detection, and the ordinate was the ion strength (count per second, cps) of ion detection, generating the map of each metabolite. The peak area of the metabolite was interpolated into the regression equation fitted by the standard curve to obtain the concentration of the metabolite. On the basis of the parameters such as sampling and/or dilution ratio, we calculated the concentration of metabolites and obtained the absolute content of each metabolite in the actual sample.

### 2.5 Intestinal flora assay

16S rRNA amplicon sequencing and analysis were conducted by OE Biotech Co., Ltd. (Shanghai, China). Total genomic DNA was extracted using a DNA Extraction Kit following the manufacturer’s instructions. The quality and quantity of DNA were verified with NanoDrop and agarose gel. Extracted DNA was diluted to a concentration of 1 ng/μL and stored at 20°C until further processing. The diluted DNA was used as the template for PCR amplification of bacterial 16S rRNA genes with the barcoded primers and Takara Ex Taq (Takara). For bacterial diversity analysis, V3-V4 variable regions of 16S rRNA genes were amplified with universal primers 343F and 798R. For eukaryotic diversity analysis, variable regions of 18S rRNA genes were amplified with universal primers 817F and 1196R. Amplicon quality was visualized by gel electrophoresis, purified with AMPure XP beads (Agencourt), and amplified for another round of PCR. After purifying with the AMPure XP beads again, the final amplicon was quantified using the Qubit dsDNA assay kit. Equal amounts of purified amplicon were pooled for subsequent sequencing.

Raw sequencing data were in FASTQ format. Paired-end reads were then preprocessed using cutadapt software to detect and cut off the adapter. After trimming, paired-end reads were filtered for low-quality sequences, denoised, merged, and detected. The chimaera reads were cut off using DADA2 with the default parameters of QIIME2 (2020.11). Finally, the software produced the representative reads and ASV abundance table. The representative read of each ASV was selected using the QIIME2 package. All representative reads were annotated and blasted against the Silva database Version 138 (or Unite) (16s rDNA) using q2-feature-classifier with the default parameters.

### 2.6 Immune function assay

Eight mice were randomly divided into three groups, namely, KB, CTX, and zunyimycin C group. The KB group was intraperitoneally injected with NS, whereas all mice in the six other groups were intraperitoneally injected with CTX (100 mg/kg) once a day for 3 days. On the fourth day, all mice were intraperitoneally injected with 0.2 mL of 6% (v/v) chicken red blood cells. Meanwhile, mice in the experimental groups were given drug by gavage once a day for 15 days; otherwise, the KB and CTX groups were given normal saline by gavage for 15 days. Mice in each group were euthanized by taking blood from orbit, and the amounts of hemolysin, IL-2, IL-6, Interferon(IFN)-y, and Tumor necrosis factor (TNF)-α were measured. On the fourth day, all mice were sensitized by intraperitoneal injection of 0.2 mL of 6% chicken red blood cells, and mice blood samples were collected from orbit. After resting at room temperature for 1 h, blood samples were centrifuged at 4°C and 2000 rpm for 20 min, and the supernatant was collected and incubated for 1 h at 37°C with 250 µL of 6% (v/v) chicken red blood cell and 250 µL of 10% guinea pig serum. The reaction was stopped at 15 min in ice and then centrifuged at 4°C and 2000 rpm for 20 min. About 200 µL of supernatant was collected in 96-well plate, and the optical density value was measured at 540 nm.

### 2.7 Quantitative real-time PCR

After the behavioral experiment, the mice were anesthetized and perfused with normal saline, and the brain tissue was removed, immediately placed in liquid nitrogen, and transferred to a −80°C freezer for cryopreservation. The brain tissue was thawed from the −80°C freezer during testing. Total RNA was extracted from mouse brain tissue using Trizol reagent (TaKaRa, Osaka, Japan). The concentration and purity of total RNA were detected using a microplate reader (MOLECULAR DEVICES, CA, USA). PrimeScript™ RT Master Mix (Perfect Real Time) kit (TaKaRa, Osaka, Japan) was used to reverse transcribe RNA to cDNA. In q-PCR, PCR was performed in Cobas z 480 (Roche Applied Science, Basel, Switzerland). cDNA was analyzed using TB Green Premix Ex Taq™ (Tli RNaseH Plus) kit (TaKaRa, Osaka, Japan). GAPDH served as an endogenous control. The Ct value of each group was derived, and the relative level of mRNA was analyzed using the 2^-ΔΔCt^ method. The primer sequences used in this experiment are shown in **Table 2**. The primers were designed using the online primer design website Primer3Plus (http://www.primer3plus.com), and the online primer analysis tool NCBI blast (https://blast.ncbi.nlm.nih.gov/Blast.cgi) was used to analyze the specificity of the primers. Primers were synthesized by Sangon Bioengineering (Shanghai) Co., Ltd.

**Table 2:** Fluorescence quantitative PCR primers.

Gene	NCBI Reference Sequence	Primer sequence	Product length	Annealing Tm (°C)
*Il-1β*	NM_008361	Forward 5’-CAGGCAGGCAGTATCACTCATT-3’ Reverse 5’-CGTCACACACCAGCAGGTTATC-3’	173	59.4 60.4
*Tnf-α*	NM_001278601	Forward 5’- CCCAGACCCTCACACTCAGAT -3’ Reverse 5’- AGCCTTGTCCCTTGAAGAGAA -3’	219	58.5 58.3
*Ifn-γ*	NM_008337	Forward 5’-TATCTGGAGGAACTGGCAAAAG -3’ Reverse 5’- GACCTCAAACTTGGCAATACTCA -3’	208	58.9 58.9
*Il-4*	NM_021283	Forward 5’- AGTTGTCATCCTGCTCTTCTTTCTC -3’ Reverse 5’- TCACTCTCTGTGGTGTTCTTCGTT -3’	177	60.5 60.7
*Iba1*	NM_001361501	Forward 5’- CCAAATACAGCAATGATGAGGAT -3’ Reverse 5’- CCCCAAGTTTCTCCAGCATT -3’	132	58.7 59.1
*Creb*	NM_001037726	Forward 5’-CCACTGTAACGGTGCCAACT-3’ Reverse 5’- GCTGCATTGGTCATGGTTAATGT-3’	208	58.2 61.6
*Ppar-γ*	NM_001127330	Forward 5’-TACTGTCGGTTTCAGAAATGCC-3’ Reverse 5’- GTCAGCGGACTCTGGATTCAG-3’	197	59.6 59.5
*Mek1*	NM_008927	Forward 5’-GTCAGCGGGCAGCTCATCGA-3’ Reverse 5’- TTCCACCTGGCACCCAAACA-3’	210	66.6 64.1
*Akt*	NM_001165894	Forward 5’-CGCCTGCCCTTCTACAACCA-3’ Reverse 5’- GGCCGTGAACTCCTCATCAA-3’	297	63.3 60.5
*Gapdh*	NM_001289726	Forward 5’-GAGTGTTTCCTCGTCCCGTAGA-3’ Reverse 5’- CAACAATCTCCACTTTGCCACT-3’	114	60.8 59.4

### 2.8 Western blot

The mouse brain tissue was removed from the −80 °C freezer, thawed, and added with 500 μL of RIPA lysis buffer (Beijing Solarbio Science & Technology Co., Ltd., Beijing, China) to 100 mg of brain tissue. The samples were homogenized with a glass homogenizer and placed at room temperature 10 min. The samples were then centrifuged at 12,000 rpm for 5 min at 4°C, and the supernatant was collected. The protein concentration was detected using a BCA kit (Beijing Solarbio Science & Technology Co., Ltd., Beijing, China). Equal amounts of proteins were separated by SDS-PAGE electrophoresis and transferred to PVDF membranes, which were blocked in 5% nonfat dry milk (dissolved in TBST) for 2 h. They were then mixed with primary antibody GFAP (1:1000, BOSTER Biological Technology Co., Ltd., Wuhan, China), CD163 (1:1000, Beijing Solarbio Science & Technology Co., Ltd., Beijing, China), IL-1β (1:1000, BOSTER Biological Technology Co., Ltd., Wuhan, China), IL-6 (1:1000, Beijing Solarbio Science & Technology Co., Ltd., Beijing, China), and GAPDH (1:1000, Cell Signaling Technology, Danvers, MA, USA) and incubated overnight at 4°C. After washing the membranes three times with TBST (5 min/time), the membranes were incubated with secondary antibody with HRP diluted in TBST containing 5% nonfat milk powder for 2 h. The membranes were completely immersed in HPR substrate luminescent solution (ThermoFisher LLC. Waltham, MA, USA), and the bands were exposed to a chemiluminometer (GBox Chem3, Syngene, the UK). The exposure results were saved for subsequent analysis.

### 2.9 Statistical analysis

The data of each group were expressed as the mean ± standard deviation (mean ± SD). Statistical analysis was performed using GraphPad Prism 8 software. One-way ANOVA was used for comparison between multiple groups, and *t*-test was used for comparison between two groups. P<0.05 indicated that the difference was statistically significant.

## 3 Result

### 3.1 Nasal administration allows zunyimycin C to enter brain tissue

Zunyimycin C was not detected in the blood of mice administered intranasally, whereas zunyimycin C was detected in the blood of mice administered by gavage and tail vein. Thus, both gavage and tail vein injection facilitated the drug’s entry into the blood circulation, but nasal administration did not. In the chromatogram of the blood extract from the gavage mice, the target peak RT was 1.498, the peak height (PH) was 0.13, and the peak area (PA) was 0.445. The concentration of zunyimycin C obtained by the standard curve was 1.1904 mg/mL, and the total amount was 0.23808 mg. In the chromatogram of the blood extract of mice injected with tail vein, the target peak RT was 1.507, PH was 0.08, and PA was 0.237. The zunyimycin C concentration was 1.1903 mg/mL, and the total amount was 0.23806 mg. Zunyimycin C was detected in the brain tissue of mice administered nasally. However, no zunyimycin C was detected in the brain tissue of mice injected by gavage and tail vein. In the liquid chromatogram of the chloroform-extracted extract of mouse brain tissue after nasal administration, the target peak RT was 1.298, PH was 0.11, and PA was 0.830. The concentration of zunyimycin C was 1.1907 mg/mL. These results suggested that intranasal administration of zunyimycin C enabled zunyimycin C to exert its effects in the brain *via* entry into the olfactory or trigeminal nerves (rather than into the blood and then across the blood–brain barrier).

### 3.2 Zunyimycin C improves learning and memory impairment in AD mouse model

**Figure 2:**
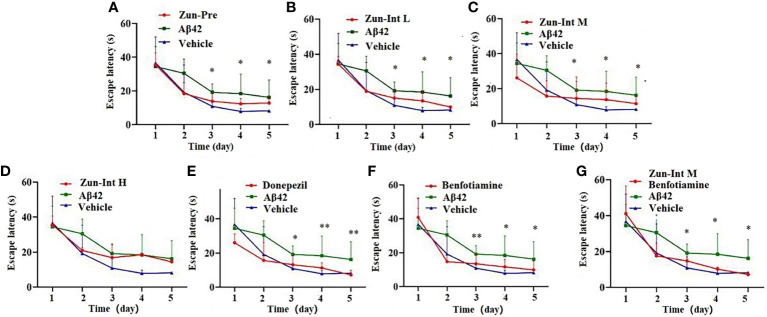
Effect of drug on the escape latency. **(A)** Zun-Pre: zunyimycin C preventive(14 mg/kg/d, intervene 21 days before modeling) effect, difference is signific after three days. **(B)** Zun-Int L: low-dose zunyimycin C (7 mg/kg/d) interventive effect, difference is signific after three days. **(C)** Zun-Int M: intermediate dose-dose zunyimycin C (14 mg/kg/d) interventive effect, difference is signific after three days. **(D)** Zun-Int H: high-dose zunyimycin C (21 mg/kg/d) interventive effect, no difference is signific after three days. **(E)** donepezil (5 mg/kg/d) interventive effect, difference is signific after three days. **(E)** donepezil (5 mg/kg/d) interventive effect, difference is signific after three days. **(F)** benfotiamine (5 mg/kg/d) interventive effect, difference is signific after three days. **(G)** benfotiamine (5 mg/kg/d) conjunction with intermediate-dose zunyimycin C (14 mg/kg/d) interventive effect, difference is signific after three days. (n=7), *P < 0.05; **P < 0.01.

The escape latency of training for 5 consecutive days in each group and the results (**Table 3** and **Figure 2**) showed that the escape latency of the benfotiamine intervention group was higher than that of the Vehicle group but lower than that of the model group (P<0.05), indicating that the cognitive ability of the benfotiamine intervention group was in the recovery period. The positive control was donepezil, their escape latency also shows the same trend as benfotiamine, i.e. lower than that of the model group (P<0.05) after three days. The escape latency of Zun-Pre group, Zun-Int L group, Zun-Int M group and Zun-Int M +benfotiamine goup are all lower than that of the model group (P<0.05).

**Table 3:** Escape latency of mice in each group.

No.	Name	Day 1	Day 2	Day 3	Day 4	Day 5
1	Zun-Pre	35.55 ± 7.01	18.43 ± 6.45	13.90 ± 4.47	12.40 ± 3.09	12.88 ± 2.83
2	Zun-Int L	34.30 ± 4.42	9.57 ± 0.75	15.01 ± 3.92	13.44 ± 5.43	9.95 ± 0.69
3	Zun-Int M	26.20 ± 13.43	15.77 ± 8.66	14.43 ± 12.32	13.74 ± 9.41	11.45 ± 4.20
4	Zun-Int H	35.56 ± 5.09	20.96 ± 1.50	16.90 ± 7.83	18.54 ± 1.02	14.44 ± 1.35
5	Donepezil	26.05 ± 5.19	15.65 ± 10.28	13.18 ± 5.49	11.30 ± 3.06	7.21 ± 2.65
6	Benfotiamine	40.86 ± 11.52	14.73 ± 3.71	13.4 ± 5.40	11.64 ± 4.41	9.95 ± 5.13
7	Benfotiamine +Zun-Int M	41.11 ± 15.41	14.27 ± 8.70	17.26 ± 4.33	10.20 ± 1.15	7.18 ± 0.90
8	Aβ42	34.47 ± 11.74	30.49 ± 8.32	19.19 ± 4.98	18.44 ± 11.53	16.22 ± 10.35
9	Vehicle	36.70 ± 15.28	19.22 ± 16.12	10.91 ± 7.77	7.89 ± 1.79	8.19 ± 0.78

**Figure 3:**
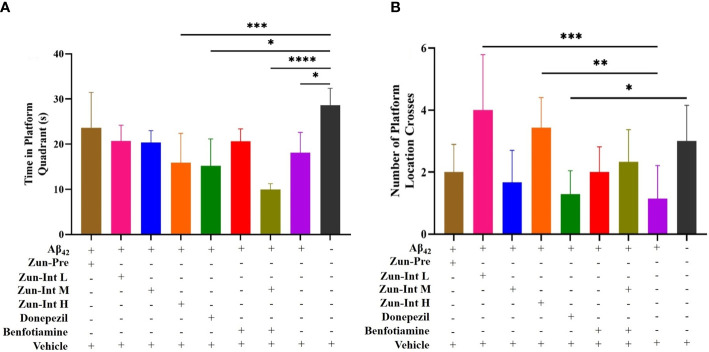
Effect of drug on the residence time in quadrant of the original platform and number of platform crossings. **(A)** Compared with AD model mice (Ab42), the residence time of the mice in the AD model group in the quadrant of the original platform was shorter than Vehicle group, the difference was statistically significant. Residence times of Zun-Int H group, donepezil group, and benfotiamine conjunction with Zun-Int M in the quadrant of the original platform were all lower than AD model group. Residence times of Zun-Pre group, Zun-Int L group, Zun-Int M group and benfotiamine intervention group longer than those in AD model group but no statistical significance. **(B)** Effect of drug on number of platform crossings. Compared with AD model mice, the platform crossing times of Zun-Int L group and Zun-Int H group were all higher than AD model model group and the difference was statistically significant.(n=7), *: P < 0.05; **: P < 0.01; ***: P < 0.001; ****: P < 0.0001.

The stay time of the mice in each group in the quadrant of the original platform (**Figure 3A**) showed that the stay time of the mice in the model group in the quadrant of the original platform was 18.08 ± 4.52 s, which was shorter than the stay time of the mice in the Vehicle group (28.65 ± 3.71 s). The difference was statistically significant (P<0.05). The residence times of the Zun-Int H group, donepezil intervention group, and combination group in the quadrant of the original platform were 15.88 ± 6.50, 15.19 ± 5.93, and 9.93 ± 1.30 s, respectively, which were all lower than that of the model group. The mice in the Zun-Pre group, Zun-Int L group, Zun-Int M group, and benfotiamine intervention group stayed longer than those in the model group in the quadrant where the original platform was located but shorter than those in the Vehicle group. These mice were in the recovery phase.

The platform crossing times of the mice in each group in the space exploration experiment (**Figure 3B**) showed that the platform crossing times of the Zun-Int L group were 4 ± 1.78, whereas that of the Zun-Int H group was 3.42 ± 0.97. These values were all higher than those of the model group. The number of platform crossings was 1.14 ± 1.06, and the difference was statistically significant (P<0.05).

**Figure 4:**
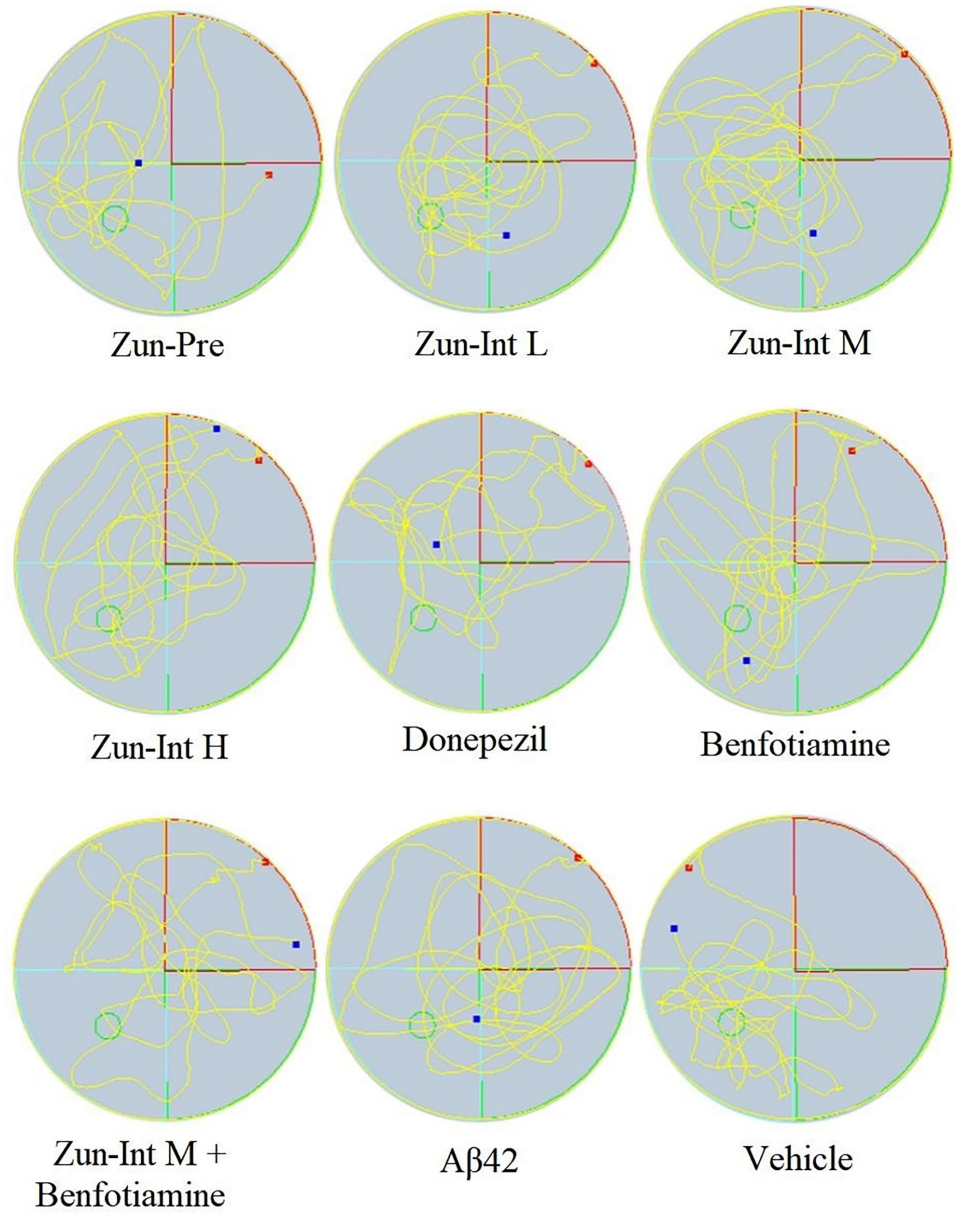
Experimental trajectory of spatial exploration of mice in different treatment groups. Compared with AD model mice (Aβ42), activities in the original platform location were more frequent and the time stay in the quadrant in the Vehicle group, Zun-Pre group, Zun-Int L group, Zun-Int M group, and benfotiamine group (P < 0.05). The red dots represent the starting position of mouse swimming, and the blue dots represent the swimming end position of mice.

From the movement trajectories of mice in the space exploration experiment (**Figure 4**), activities in the original platform location were more frequent in the Vehicle group, Zun-Pre group, Zun-Int L group, Zun-Int M group, and benfotiamine intervention group. Mice in the model group, donepezil intervention group, Zun-Int H group, and combination group demonstrated irregular movements in the pool, and they did not stay in the quadrant where the original platform was located for a long time.

### 3.3 High-dose zunyimycin C can reduce the mRNA expression of neuroinflammatory factors in brain tissue of AD mouse model

**Figure 5:**
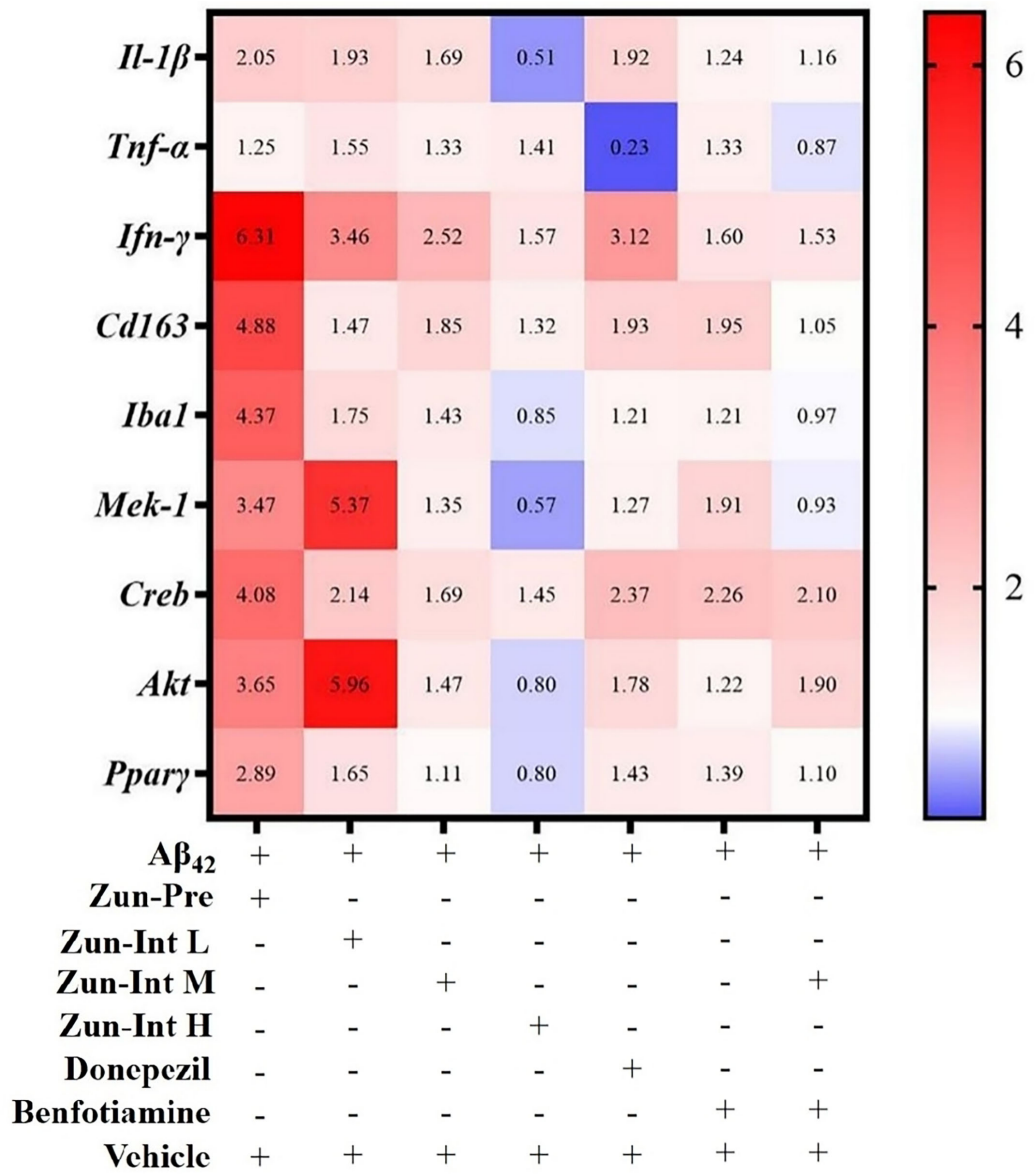
Effect of drugs on mRNA expression levels. AD model group (Aβ42) fold change was defined as 1, compared with AD model mice fold change higher than 2 was defined as upregulation and less than 0.75 as downregulation.

Differential expression of mRNAs of inflammatory factors and pathways in brain tissue of the AD mouse model after different interventions was compared with the model group (**Figure 5**). The model group fold change was defined as 1. The target gene expression fold change was greater than 2 as upregulation and less than 1 as downregulation. The results showed that the mRNA expression levels of the Il-1β, IFN-γ, Cd163, Iba1, Mek-1, Creb, Akt, and Pparγ genes in the brain tissue of the mice in the Zun-Pre group were all upregulated. The mRNA expression levels of IFN-γ, Mek-1, Creb, and Akt genes in the brain tissue of mice in the low-dose intervention group were upregulated. The mRNA expression of the IFN-γ gene in the brain tissue of the mice in the medium-dose zunyimycin intervention group was upregulated. The mRNA expression levels of Il-1β, Iba1, Mek-1, Akt, and Pparγ in the brain tissue of the mice in the Zun-Int H were all downregulated. The mRNA expression of the Tnf-α gene in the brain tissue of mice in the donepezil group was downregulated, whereas the mRNA expression of IFN-γ and Creb gene was upregulated. The mRNA expression of the Creb gene in the brain tissue of the mice in the benfotiamine group was upregulated. The mRNA expression levels of the Tnf-α, Iba1, and Mek-1 genes in the brain tissue of the mice in the combination group were downregulated, and the mRNA expression of the Creb gene was upregulated.

### 3.4 Effects of zunyimycin C on the expression of inflammation-related proteins in AD mouse model

**Figure 6:**
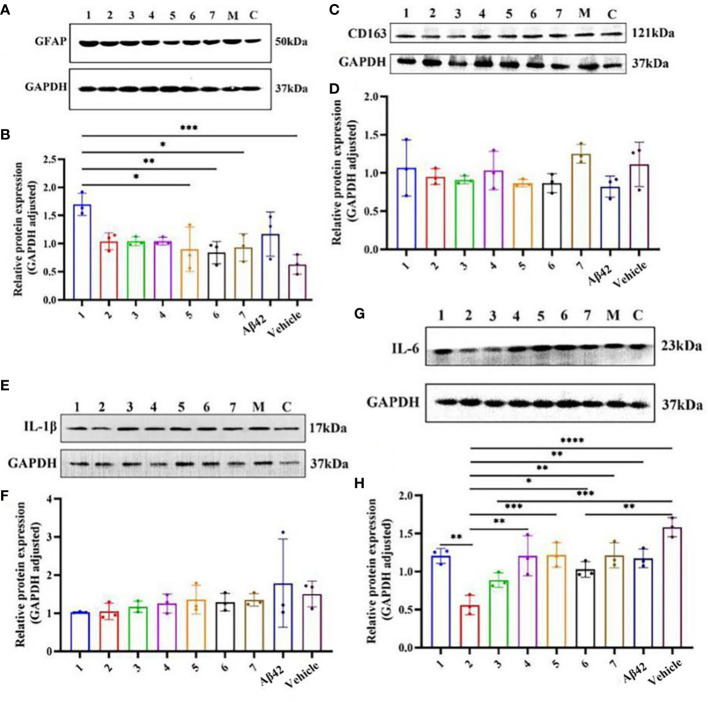
Effects of drugs on the expression of inflammation-related proteins in brain tissue of AD mouse model. **(A, B)** Protein expression level GFAP. **(C, D)** Protein expression level CD163. **(E, F)** Protein expression level Il-1β. **(G, H)** Protein expression level Il-6. 1:Zun-Pre; 2:Zun-Int Ll; 3: Zun-Int M; 4: Zun-Int H; 5: Donepezil; 6: Benfotiamine; 7:Zun-Int M+Benfotiamine; M:AD mouse model (Aβ42); C:vehicle. *:*P < 0.05*, **:*P < 0.01*, ***:*P < 0.001*; ****P < 0.0001.

The difference in the expression of inflammation-related proteins in the brain tissue of AD mouse models after different interventions (**Figure 6**) showed that the relative expression of GFAP in the brain tissue of the AD mouse model in the Zun-Pre group was higher than that in the donepezil group, benfotiamine group, Zun-Int M combined benfotiamine group, and Vehicle group; the difference was statistically significant (P<0.05). There was no significant difference in the relative expression of GFAP among different doses of zunyimycin C intervention groups. The relative expression of GFAP in the donepezil group, benfotiamine group, and combination group was lower than that in the zunyimycin C intervention group, but the relative expression of GFAP in these intervention groups was lower than that of the model group and higher than that of the Vehicle group. The relative expression of IL-6 protein in the brain tissue of mice in the Zun-Int L group was significantly lower than that in the Zun-Pre group, Zun-Int H, donepezil group, benfotiamine group, combination group, model group, and Vehicle group. The difference was statistically significant (P<0.05).

#### 3.4.1 Effects of zunyimycin on the biosynthesis of intestinal SCFAs

Differences in the composition and content of SCFAs were calculated (**Table 4**) and the there was no significant difference in the content of acetic acid in zunyimycin C group compared with the CTX, indicating that the zunyimycin C group did not affect the recovery of the intestinal acetic acid level in the immunocompromised mice. The level of propionic acid in zunyimycin C-treated mice could all be restored to the level in immunocompromised mice (P<0.05), and the levels of isobutyric acid and valproic acid in the zunyimycin C group were significantly lower than that in the CTX group (P<0.05). The hexanoic acid content in the zunyimycin C group was 0, suggesting that the drug inhibited the synthesis of hexanoic acid of intestinal flora, whereas zunyimycin C did not affect the recovery of isobutyric acid and isovaleric acid.

**Table 4:** SCFAs in intestinal tract of mice.

ID	Metabolites	Vehicle	CTX	Zunyimycin C
1	Acetic Acid	2349158.9	592236.3	728426.4
2	Butyric Acid	886153.1	152134.6	25477.3
3	Hexanoic Acid	1063.6	213.8	0
4	Isobutyric Acid	35617.9	4936.5	5732.2
5	Isovaleric Acid	29310.1	6913.759	6339.1
6	Pentanoic Acid	30851.4	7092.05	4455.8
7	Propionic Acid	458855.6	44607.2	83800.0

#### 3.4.2 Effects of zunyimycin on intestinal flora diversity

**Figure 7:**
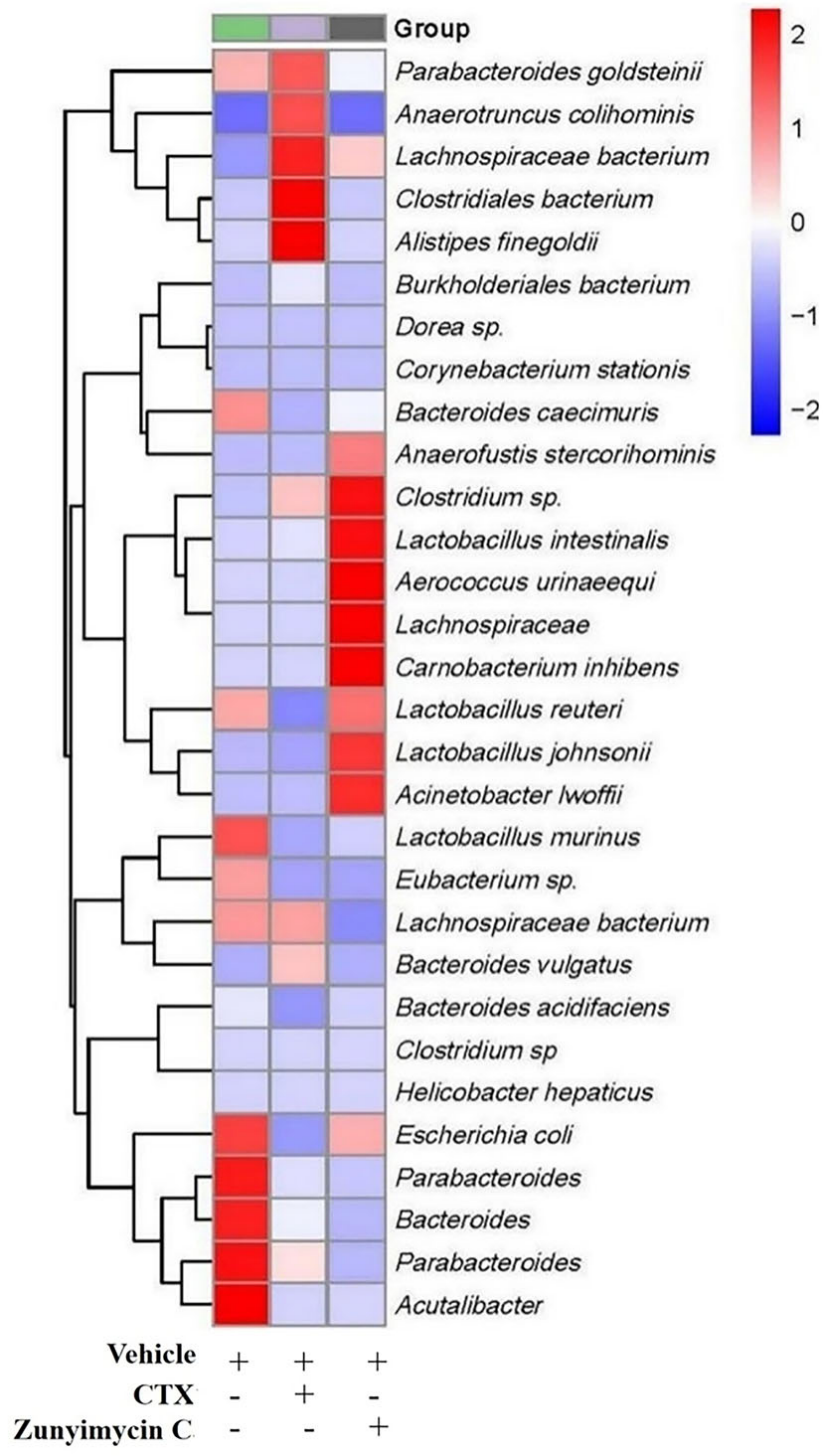
Effect of zunyimycin on intestinal flora of mice of mice. The intestinal bacteria in the zunyimycin C group changed greatly at the species level. The species abundance of the bacteria that accounted for the most of the total number of bacteria was arranged from high to low, the community structure of different groups of mice varied at the genus level. Compared with AD model mice, change of relative abundance of intestinal flora higher than 1 was defined as upregulation and lower than -1 as downregulation.

Analysis of the abundance of intestinal bacteria at the genus level of mice with different treatments showed (**Figure 7**) that the intestinal bacteria in the zunyimycin C group changed greatly at the species level. The species abundance of the bacteria that accounted for the most of the total number of bacteria was arranged from high to low, as follows: *Acteroides acidifaciens*, *Lactobacillus murinus*, *Lactobacillus johnsonii*, *Clostridiales*, *Lactobacillus reuteri*, *Lactobacillus intestinalis*, *Lachnospiraceae*, *Bacteroides caecuris*, *Parabolides gordonii*, *Parabolides goldsteinii*, *Parabolides distasonis*, *Bacteroides stercorosoris*, *Burkholderiales*, *Lachnospiraceae*, *Eubacterium*, *Escherichia coli*, *Dorea*, *Corynebacterium stationis*, *Lachnospiraceae*, *Anaerotruncus colihominis*, *Clostridium*. *Roseburia*, *Carnobacterium inhibiens*, *Acinetobacter lwoffii*, *Clostridium*, *Bacteroides vulgatus*, *Anaerofusis stereohominis*, *Acutalibacter muris*, *Aerococcus urinaeequi*, *Helicobacter hepaticus*, and *Alistipes finegoldii*. As shown in **Figure 7**, the community structure of different groups of mice varied at the genus level. The dominant flora of the zunyimycin C group were *Clostridium*, *Lactobacillus*, *Aerococcus urinaeequi*, *Lachnospiraceae*, *Carnobacterium*, and *Acinetobacter*.

#### 3.4.3 Effect of drugs on hemolysin levels

**Figure 8:**
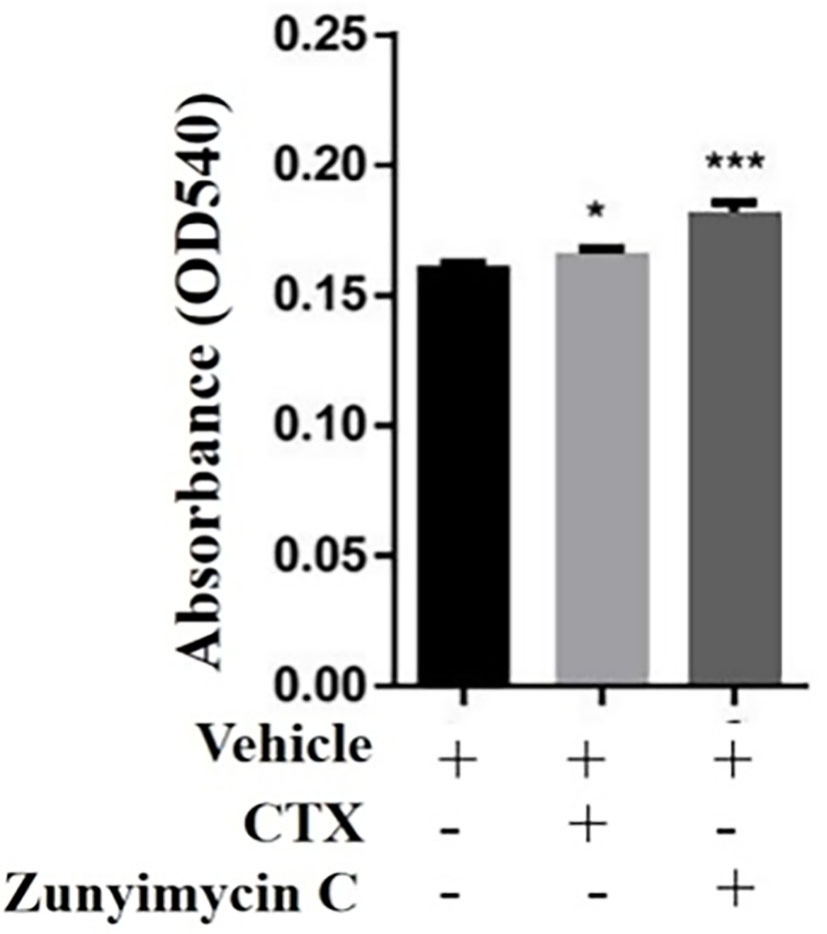
Effect of drugs on hemoilsin levels. KB, blank control group, CTX:model group;Zun C, zunyimycin C group; *: P < 0.05;***: P < 0.001.

Hemolysin levels were detected. Compared with the CTX group, hemolysin increased significantly in the zunyimycin C groups (P<0.001), indicating that the humoral immunity of mice could be restored to a certain extent by zunyimycin C (**Figure 8**).

#### 3.4.4 Effects of zunyimycin on serum cytokines

**Figure 9:**
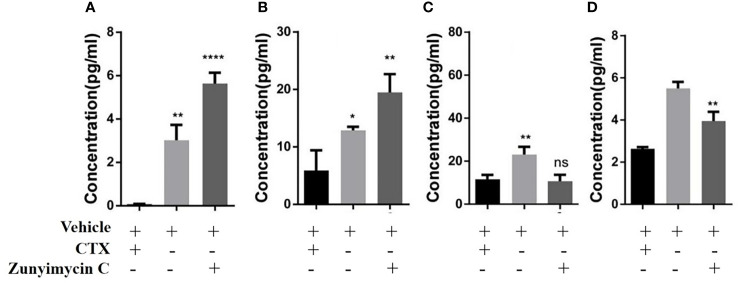
Effect of drugs on cytokine in mice. **(A)** the level of IL-2. **(B)** the level of IL-6. **(C)** the level of IFN-γ. **(D)** the level of TNF-α l. *P < 0.05; **P < 0.01; ****P < 0.0001; ns, No statistical difference.

Effects of zunyimycin on serum cytokines were detected, and the results (**Figure 9**) demonstrated that the level of IL-2 in the CTX group decreased significantly compared with that in the KB group (P<0.01) and was restored after treatment with zunyimycin C (P<0.05). Meanwhile, the level of IL-6 in the CTX group was significantly lower than that in the KB group (P<0.05) and increased significantly after treatment with the drug (P<0.05). The level of IFN-γ in the CTX group was significantly lower than that in the KB group (P<0.0001), and no significant (ns) change was found after treatment with zunyimycin C. Serum TNF-α levels increased after treatment with zunyimycin C (P<0.01).

## 4 Discussion

In this experiment, zunyimycin C was detected in the brain tissue of mice administered nasally but not in the blood. Thus, zunyimycin C enters the brain tissue through nerves after entering the nasal cavity, but which nerve and which way it mainly enters the brain are unclear, and the distribution of the drug in the brain is also unknown (Illum, 2003). Research suggested that the nasal-to-brain pathway *via* the olfactory and trigeminal nerves may involve paracellular, transcellular, and neuronal transport. At this time, the drug is not absorbed by the blood vessels in the nasal cavity and enters the bloodstream. Less drugs enter the blood circulation and cannot be detected by the instrument. In the previous study of the research group, after zunyimycin C treated Sprague-Dawley rats infected with MRSA, we found that the liver of the rats was congested, the spleen was congested, and small abscess nodules were found on the surface of the kidney, that is, zunyimycin C had liver, kidney, and lung toxicity (Wang, 2018). Therefore, systemic administration *via* blood transport has certain side effects. No visible damage to other organs was found in mice administered nasally in the experiment, which proved the safety of intranasal administration. Zunyimycin C in the brain tissue of mice administered nasally was 11.81% of the administered dose. At this time, the concentration of the drug was related to the absorption of the drug by the nasal epithelial nerves of the mice, the resistance of the mice to the loss of the drug during the administration process, and the extraction efficiency. Among them, the absorption of drugs by the nasal epithelium is the most difficult to control.

AβO is cytotoxic; after injection into the lateral ventricle, it is transmitted to the entire brain tissue along with the cerebrospinal fluid, causing neuroinflammation and neuronal cell death. Its accumulation in the hippocampus can lead to impaired memory function (Lee et al., 2017; Hu et al., 2022). The mechanism of action of donepezil is that the anion binding site on its indole ring binds to acetylcholine esterase (AChE), thereby reducing the activity of AChE, increasing the content of acetylcholine in the brain, improving the efficiency of nerve signal transmission, and enhancing the cognitive ability of patients. However, donepezil does not fundamentally treat AD; it cannot prevent the occurrence and development of AD and can only temporarily relieve symptoms. The results showed that the escape latency of mice in the Zun-Pre group, Zun-Int L group, and Zun-Int M group was shorter than that in the model group. The dwell time in the quadrant of the original platform was longer than that of the model group. The number of platform crossings was more than that of the model group. Thus, zunyimycin C improved the learning and memory ability of these three AD mouse models. This experiment also found that the escape latency of donepezil-intervened AD mice was close to that of the Vehicle group in the positioning navigation experiment. However, in the space exploration experiment, the number of platform crossings and the dwell time in the quadrant of the original platform were similar to those of the model group. This phenomenon may be because donepezil has a certain effect on the amygdala and is effective in the recovery of learning impairment in AD mouse models (Li et al., 2018; Ahmadi et al., 2021). Research has shown that the amygdala plays an important role in visual object-based reinforcement learning and other forms of reward learning (Costa et al., 2016; Taswell et al., 2021).

The results of the Zun-Int H group in the positioning navigation experiment were not ideal, but the mice in this group crossed the platform more frequently than the mice in the Vehicle group. Thus, the spatial memory ability of mice was gradually enhanced, which may be because the intervention of high-dose zunyimycin C improved the hippocampal spatial memory impairment of AD mice; this finding needs to be verified by biochemical experiments (Ahmadi et al., 2021). Studies have shown that the hippocampus and its surrounding areas in the brain are important brain areas that support spatial memory (Ren et al., 2022). Hippocampal pyramidal cells in the hippocampus have spatial firing correlations. These cells fired only in certain locations on the experimental site. After spatial cues were removed, their discharge signal at that spatial location remained. Therefore, in the memory experiment, the original position of the spatial cue could be remembered (O'keefe and Conway, 1978; Khlaifia et al., 2022).

The experimental results showed that the mRNA expression levels of Il-1β, IFN-γ, Cd163, and Iba1 in the brain tissue of AD mice in the Zun-Pre group were upregulated compared with those in the model group, which indicated that the number of microglia in the brain tissue of the mice in this group was upregulated. This finding resulted in the excessive activation of inflammatory factor-encoding genes Il-1β and IFN-γ and compensatory upregulation of CD163 for the protection of nerve cells. The mRNA expression of Iba1 in the brain tissue of the mice in the Zun-Int H was downregulated, indicating that the number of microglia in the brain tissue of the mice in this group was reduced. This phenomenon may be the reason for the downregulated mRNA expression of Il-1β in the brain tissue of this group of mice. Studies have shown that chronic inflammatory environment in the brain can allow peripheral NK cells to enter the brain (Solana et al., 2018). Peripheral NK cells entering the brain are activated by cytokines secreted by microglia and produce IFN-γ and TNF-α. The experimental results showed that the mRNA expression of IFN-γ in the brain tissue of mice in the Zun-Pre group, Zun-Int L group, Zun-Int M group, and donepezil group was upregulated, which may be related to the entry of peripheral NK cells into the brain.

There is growing evidence that some natural products can play a neuroprotective role by activating the PI3K/AKT pathway, turning on or off downstream regulatory proteins, including mTOR and GSK3 (Long et al., 2021). Research found that Aβ is engulfed by microglia through the cell surface receptor TREM2 of microglia. TREM2 interacts with the adaptor proteins DAP12 and DAP10 (Atagi et al., 2015). They can transmit cell signals by activating the PI3K-AKT-mTOR signaling pathway, inhibiting the PI3K-AKT-mTOR pathway to promote autophagy, and activating PI3K-AKT-mTOR to inhibit autophagy (Neill, 2013; Ji et al., 2022). TLR4 activated on microglia can activate the Ras Raf MEK-ERK signaling pathway to increase NF-κB nuclear translocation and enhance the coding of inflammatory cytokine (TNF-α and Il-1β) gene expression (Fang et al., 2017; Willis et al., 2020; Wen et al., 2022). The experimental results showed that the mRNA expression levels of Mek-1, Creb, Akt, and Pparγ in the brain tissue of mice in the Zun-Pre group were upregulated. The mice in this group were given prophylactic administration for 3 weeks before the modeling operation. At this time, prophylactic administration of zunyimycin C could not prevent the inflammatory damage caused by Aβ_1-42_ oligomers to mouse brain tissue. Intracranial injection of Aβ_1-42_ oligomers formed Aβ plaques in brain tissue. Mouse brain tissue blocked the clearance of Aβ. Aβ bound to TREM2 on the surface of microglia and was phagocytosed. The PI3K-AKT-mTOR signaling pathway was overactivated. Autophagy was inhibited, thereby promoting cell survival. At the same time, Aβ_1-42_ oligomers activated the Ras-Raf-MEK-ERK signaling pathway to make microglia secrete inflammatory factors. The mRNA expression levels of Mek-1 and Akt were upregulated in the brain tissue of mice in the Zun-Int L group. We speculated that zunyimycin C may participate in the immune response by activating the Ras-Raf-MEK-ERK signaling pathway to stimulate microglia to produce more inflammatory factors. At the same time, zunyimycin C inhibited autophagy and promoted cell survival by activating the PI3K-AKT-mTOR signaling pathway. The Zun-Int H group was opposite to the low-dose zunyimycin C group. This result echoed the behavioral results, but the reasons for these findings need further experiments for verification. In addition, based on the relationship between PI3K/Akt signaling pathway and AD, previous studies have shown that an alkaloid BBR can reduce the Aβ levels, glial activation and cognitive impairment in the TgCRND8 mouse model by activating PI3K/Akt/GSK3 signaling pathway. The researchers further explored and found that its mechanism is related to the reduction of CTFs and p-APPs levels (Durairajan et al., 2012). All these evidences indicate that AD is closely related to the PI3K/Akt pathway as well as the genes and proteins downstream of this pathway, but the more detailed mechanism needs to be further studied.

The expression level of GFAP in the brain tissue of AD model mice in the Zun-Pre group was significantly higher than that in the other intervention groups. This result indicated that the number of mature astrocytes in the brain tissue of the mice in this group significantly increased. The mice in this group were tested after 8 days of modeling. At this time, the mouse brain tissue was in an environment of neuroinflammation caused by Aβ. Thus, the prophylactic administration of zunyimycin C did not prevent acute neuroinflammation in AD mice. The experimental results demonstrated that the relative expression of CD163 protein in the brain tissue of mice in the model group was the lowest, and that of the combination group was the highest. CD163 is a phenotypic marker of M2c microglia, which can turn off microglia-mediated immune responses. The relative expression of CD163 protein in the brain tissue of the AD mouse model in the other intervention groups was between the model group and the Vehicle group, indicating that zunyimycin C intervention had no effect on the activation of M2c-type microglia in the mouse brain. The relative expression of IL-6 in the brain tissue of mice in the Zun-Int L group was lower than that in the other intervention groups, and the difference was statistically significant (P<0.05). We speculated that zunyimycin C promoted the clearance of Aβ in the brain tissue and reduced the expression of IL-6 in the brain tissue, thereby improving the long-term memory of AD mice, which was consistent with the behavioral results.

## 5 Conclusion

Combined with the results of behavioral, real-time quantitative PCR, and Western blot experiments, we found that the intervention of low-dose zunyimycin C improved the learning and memory impairment of our AD mouse model. The relative expression levels of GFAP, IL-1β, and IL-6 were lower than those in the model group. On the one hand, zunyimycin C may promote the clearance of Aβ and reduce the activation of M1-type microglia and A1-type astrocytes caused by Aβ deposition. On the other hand, zunyimycin C may mediate the neuroprotective effects of reactive microglia and reactive astrocytes *via* the PI3K-AKT-mTOR and Ras-Raf-MEK-ERK pathways. The secretion of inflammatory factors such as IL-1β and IL-6 attenuated neuroinflammation in AD mice and led to behavioral improvement. Zunyimycin C may play a role in immunological enhancement by changing intestinal flora diversity and SCFAs.

## Data availability statement

The data presented in the study are deposited in the SRA repository, accession number PRJNA897655.

## Ethics statement

The animal study was reviewed and approved by the Medical Biological Research Ethics Committee of Medical School of Yan ‘an University.

## Author contributions

CY and YL designed the study and provided the research funding. XW, ZL, XL and YY performed the chemistry experiments. XW, RG and XC performed the molecular biological experiments and cell biological experiments and prepared the manuscript. XW, WL, RG, XH, HQ, CL, RS, BL and ZW performed the animal experiment. All authors contributed to the article and approved the submitted version.
